# Association Between Risk Factors and Major Cancers: Explainable Machine Learning Approach

**DOI:** 10.2196/62833

**Published:** 2025-05-02

**Authors:** Xiayuan Huang, Shushun Ren, Xinyue Mao, Sirui Chen, Elle Chen, Yuqi He, Yun Jiang

**Affiliations:** 1 Department of Biostatistics, Yale University, New Haven, CT, United States; 2 School of Nursing, University of Michigan–Ann Arbor, 400 North Ingalls Street, Ann Arbor, MI, 48109, United States, 1 7347633705, 1 7346472416; 3 College of Literature Science and the Arts, University of Michigan–Ann Arbor, Ann Arbor, MI, United States; 4 University Library, San Jose State University, San Jose, CA, United States

**Keywords:** electronic health record, EHR, cancer risk modeling, risk factor analysis, explainable machine learning, machine learning, ML, risk factor, major cancers, monitoring, cancer risk, breast cancer, colorectal cancer, lung cancer, prostate cancer, cancer patients, clinical decision-making

## Abstract

**Background:**

Cancer is a life-threatening disease and a leading cause of death worldwide, with an estimated 611,000 deaths and over 2 million new cases in the United States in 2024. The rising incidence of major cancers, including among younger individuals, highlights the need for early screening and monitoring of risk factors to manage and decrease cancer risk.

**Objective:**

This study aimed to leverage explainable machine learning models to identify and analyze the key risk factors associated with breast, colorectal, lung, and prostate cancers. By uncovering significant associations between risk factors and these major cancer types, we sought to enhance the understanding of cancer diagnosis risk profiles. Our goal was to facilitate more precise screening, early detection, and personalized prevention strategies, ultimately contributing to better patient outcomes and promoting health equity.

**Methods:**

Deidentified electronic health record data from Medical Information Mart for Intensive Care (MIMIC)–III was used to identify patients with 4 types of cancer who had longitudinal hospital visits prior to their diagnosis presence. Their records were matched and combined with those of patients without cancer diagnoses using propensity scores based on demographic factors. Three advanced models, penalized logistic regression, random forest, and multilayer perceptron (MLP), were conducted to identify the rank of risk factors for each cancer type, with feature importance analysis for random forest and MLP models. The rank biased overlap was adopted to compare the similarity of ranked risk factors across cancer types.

**Results:**

Our framework evaluated the prediction performance of explainable machine learning models, with the MLP model demonstrating the best performance. It achieved an area under the receiver operating characteristic curve of 0.78 for breast cancer (n=58), 0.76 for colorectal cancer (n=140), 0.84 for lung cancer (n=398), and 0.78 for prostate cancer (n=104), outperforming other baseline models (*P*<.001). In addition to demographic risk factors, the most prominent nontraditional risk factors overlapped across models and cancer types, including hyperlipidemia (odds ratio [OR] 1.14, 95% CI 1.11‐1.17; *P*<.01), diabetes (OR 1.34, 95% CI 1.29‐1.39; *P*<.01), depressive disorders (OR 1.11, 95% CI 1.06‐1.16; *P*<.01), heart diseases (OR 1.42, 95% CI 1.32‐1.52; *P*<.01), and anemia (OR 1.22, 95% CI 1.14‐1.30; *P*<.01). The similarity analysis indicated the unique risk factor pattern for lung cancer from other cancer types.

**Conclusions:**

The study’s findings demonstrated the effectiveness of explainable ML models in assessing nontraditional risk factors for major cancers and highlighted the importance of considering unique risk profiles for different cancer types. Moreover, this research served as a hypothesis-generating foundation, providing preliminary results for future investigation into cancer diagnosis risk analysis and management. Furthermore, expanding collaboration with clinical experts for external validation would be essential to refine model outputs, integrate findings into practice, and enhance their impact on patient care and cancer prevention efforts.

## Introduction

Cancer is a life-threatening disease and leading cause of death worldwide. In 2024, 611,000 people were estimated to have died from cancer in the United States, and the estimated new cancer cases will reach more than 2 million for the first time [1]. This surge includes rising incidence rates for major cancers, including breast, prostate, lung, and colorectal cancers, which display the trend of increasingly affecting younger individuals who have many more years of life expectancy [1]. The US Preventive Services Task Force modified the recommended age for colorectal cancer screening from 50 to 45 years for people at average risk in 2021 and adjusted the recommendation for breast cancer screening for all women to start at the age of 40 years in 2024 [2,3]. Similar upward trends in the incidence of early-onset cancers are observed in other high-income countries, suggesting shared risk factors and exposures across these regions. However, besides those uncontrollable risk factors, such as previous cancer diagnosis, family history of cancer, and genetics or inherited cancer syndrome, many cancer risk factors, including lifestyle factors, are modifiable and can be managed to decrease people’s risk for cancer [4].

Extensive evidence highlights the potential benefits of early identification of individuals at high risk for cancer, which can contribute to improved prevention, more effective treatment, reduced cancer burden, and better long-term outcomes. However, demonstrating a clear survival advantage [5] from screening remains challenging, with notable exceptions such as cervical cancer [6]. It is essential to address biases like lead-time and length bias in screening, as they can overestimate its benefits, ensuring accurate evaluations [7]. In the context of breast cancer, it was estimated that early access to treatment services following breast cancer screening could have reduced breast cancer mortality by 25%‐40% [8]. Given the tremendous benefits of early identification of high-risk patients, an increasing number of cancer risk prediction models have been developed [9]. However, Traditional models used for cancer risk prediction, such as logistic regression (LR) and Cox regression, often demonstrate moderate discrimination accuracy, with an area under the receiver operating characteristic curve (AUC) ranging from 0.53 to 0.64 [10-13]. These models frequently emphasize family history and may have limited generalizability, potentially introducing biases when applied to specific subpopulations [14,15]. Furthermore, nontraditional risk factors, such as chronic diseases, are often overlooked, despite evidence suggesting that chronic conditions can elevate cancer risk similarly to lifestyle factors [16]. This highlights the need for more advanced methods to enhance cancer diagnosis risk prediction and support effective cancer prevention strategies.

Machine learning has shown promising potential in cancer prediction by leveraging electronic health record (EHR) data to identify risk factors [17]. Current applications range from developing predictive models for early cancer detection to personalized treatment recommendations and outcome predictions, based on various patient characteristics and biomarkers. Despite these advancements, several challenges remain in cancer prediction using machine learning [18]. A key issue is the need for a deeper understanding of risk factors within and across different cancer types [19]. As research progresses, explainable machine learning offers a meaningful step forward in improving the efficacy and transparency of predictive models [20-22]. These models not only enhance predictive accuracy but also provide interpretable insights into how predictions are made, fostering trust and facilitating clinical decision-making [23]. By systematically identifying and excluding irrelevant features, explainable approaches can reduce noise and streamline the prediction process. However, it is important to recognize that feature selection algorithms can be sensitive to dataset characteristics, where small changes in the data may lead to differing results [24]. This underscores the importance of carefully selecting features that are most relevant, contributing to a deeper understanding of cancer diagnosis risk factors and improving predictive performance.

Hence, this study presented comprehensive research aimed at uncovering the association between pivotal factors and the risks of 4 major cancer diagnoses (breast, prostate, lung, and colorectal) through the use of explainable machine learning techniques on penalized LR, random forest (RF), and multilayer perceptron (MLP). Our primary objective was to pinpoint the significant features that exert an influence on the risks associated with the diagnosis of these major cancers and to delineate the patterns of risk factors corresponding to each cancer type. Such insights can contribute to enhanced risk monitoring and patient stratification and provide valuable support for clinicians in their decision-making processes, ultimately improving the quality patient care. By elucidating these critical factors and their associated risk factor patterns, we provided clinicians valuable insights through rigorous analysis for enhancing risk monitoring and patient care across various cancer types.

## Methods

### Experimental Dataset

Our study was conducted using data from Medical Information Mart for Intensive Care (MIMIC)–III, a comprehensive, structured, longitudinal EHR dataset that is publicly available [25]. This dataset contains deidentified, detailed clinical data from intensive care unit (ICU) admissions between 2001 and 2012 at Beth Israel Deaconess Medical Center in Boston, Massachusetts, and is accessible to the global research community under a data use agreement. We used the most recent version (v2.0 released in January 2023) for this work which contains a broad spectrum of data, including information on individual patients’ health and health care from various inpatient and outpatient visits, such as diagnoses, prescriptions, lab tests, and procedures. These visits include emergency room admissions and subsequent hospital transfers, where a patient’s transfer to a ward or subsequent re-admission to the ICU within the same hospitalization period was considered a single visit. In total, this dataset contains 58,976 admissions of 46,520 patients.

Additionally, we investigated the health status and prevalence of a few common chronic diseases for the MIMIC-III dataset, compared with the prevalence of these chronic diseases in the US population. The MIMIC-III dataset shows that hypertension affects 47.97% of ICU patients, while in the US population, prevalence ranges from 46.9% to 49.4% [26,27]. Diabetes mellitus is present in 21.20% of MIMIC-III patients, whereas it affects 11.6% of the US population and 14.7% of adults [28]. Hypercholesterolemia appears in 14.94% of ICU cases, with US estimates between 10% and 11.4% [29,30]. Congestive heart failure is recorded in 27.38% of MIMIC-III patients, while the lifetime risk in the US is 24% [31]. Esophageal reflux affects 15.33% of ICU patients and 20% of people in the US [32]. Pneumonia is diagnosed in 12.46% of ICU patients, while 24.9% of US adults have reported cases [33]. Anemia affects 14.02% of ICU patients, while 5.6% of the US population has the condition [34]. Acquired hypothyroidism is observed in 10.71% of MIMIC-III patients and 4.6% of US adults [35]. Tobacco use is recorded in 7.76% of ICU cases, while 19.8% of US adults report smoking [36]. Depressive disorders affect 8.17% of ICU patients, while 9.5% of American adults have been diagnosed [37]. Chronic airway obstruction is reported in 10.24% of MIMIC-III cases, while national estimates range from 6.0% to 6.1% [38].

### Data Preprocessing

We included patients with 4 types of cancers (breast, colorectal, lung, and prostate) identified using *International Classification of Diseases, Ninth Revision* (*ICD-9*) codes associated with the diagnosis of each type of cancer (Table S1 in Multimedia Appendix 1).

**Figure 1.**
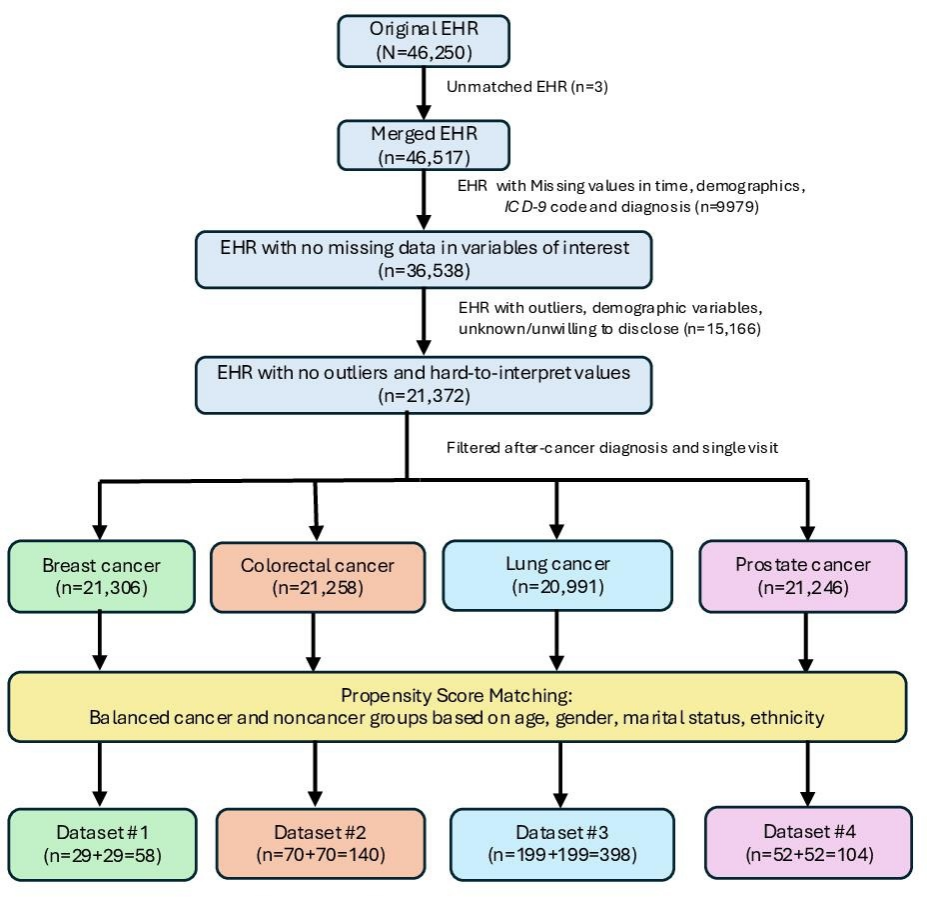
Medical Information Mart for Intensive Care (MIMIC)–III data processing pipeline. EHR: electronic health record; *ICD-9*: *International Classification of Diseases, Ninth Revision*.

We took a few steps to preprocess the experimental dataset, starting with the consolidation of 3 main tables from the MIMIC-III database. These included: (1) foundational patient information, capturing demographics and initial hospital admission data; (2) a reference table for *ICD-9* codes, detailing both codes and corresponding diagnostic labels; and (3) logs of patient visit sequences with associated *ICD-9* codes. This consolidation linked the records via patient IDs to construct a detailed longitudinal dataset. Figure 1 illustrates the data processing workflow of this study. Patients’ ages were determined by deducting their date of birth from their initial hospital admission date, with the result rounded to the nearest year. Any patient records missing demographic details (such as ethnicity, marital status, or religion) were omitted, narrowing the dataset to a total of 21,372 unique individuals. Our study focused on patients who had multiple hospital visits prior to their cancer diagnosis presence in the record to identify potential risk factors. After a cancer diagnosis code was recognized, further visits were disregarded. These records were combined with those of patients without a cancer diagnosis. A label was created as 1 if a visit included an *ICD-9* code for a cancer diagnosis and 0 if not. To ensure a balanced dataset in terms of cancer diagnosis, the study matched patients diagnosed with cancer with those without cancer using propensity score matching based on demographic factors. Table 1 contains a detailed description of patient characteristics for 4 cancer types.

**Table 1.** Characteristics of patients for 4 types of cancer.

	Breast cancer (n=58)		Colorectal cancer (n=140)		Lung cancer (n=398)		Prostate cancer (n=104)	
	With cancer (n=29)	Without cancer (n=29)	With cancer (n=70)	Without cancer (n=70)	With cancer (n=199)	Without cancer (n=199)	With cancer (n=52)	Without cancer (n=52)
Age (years), median (range)	60 (40‐86)	60 (39‐86)	76 (21‐87)	75.5 (29-87)	69 (39‐88)	69 (39‐87)	74.5 (52-88)	73.5 (52-88)
Sex, n (%)								
Female	27 (93.1)	27 (93.1)	35 (50.0)	33 (47.1)	93 (46.7)	83 (41.7)	0 (0)	0 (0)
Male	2 (6.9)	2 (6.9)	35 (50.0)	37 (52.9)	106 (53.3)	116 (58.3)	52 (100)	52 (100)
Race, n (%)								
White	20 (69.0)	21 (72.4)	51 (72.9)	52 (74.3)	162 (81.4)	157 (78.9)	41 (78.8)	39 (75.0)
Non-White	9 (31.0)	8 (27.6)	19 (27.1)	18 (25.7)	37 (18.6)	42 (21.1)	11 (21.2)	13 (25.0)
Marital status, n (%)^a^								
Married	16 (55.2)	15 (51.7)	37 (52.9)	41 (58.6)	109 (54.8)	113 (56.8)	31 (59.6)	32 (61.5)
Not married	13 (44.8)	14 (48.3)	33 (47.1)	29 (41.4)	90 (45.2)	86 (43.2)	21 (40.4)	20 (38.5)
Religion, n (%)								
Catholic	15 (51.7)	13 (44.8)	35 (50.0)	31 (44.3)	111 (55.8)	107 (53.8)	19 (36.5)	19 (36.5)
Jewish	7 (24.1)	7 (24.1)	17 (24.3)	18 (25.7)	33 (16.6)	31 (15.6)	11 (21.2)	10 (19.2)
Protestant Quaker	7 (24.1)	7 (24.1)	14 (20.0)	11 (15.7)	42 (21.1)	38 (19.1)	16 (30.8)	17 (32.7)
Other	0 (0)	3 (10.3)	4 (5.7)	10 (14.3)	13 (6.5)	23 (11.6)	6 (11.5)	6 (11.5)
ICU^b^ visits, n								
Mean	2.5	1.5	2.6	1.5	2.6	1.6	2.5	1.5
Maximum	5	5	6	6	10	12	7	5
Minimum	2	1	2	1	2	1	2	1
*ICD-9*^c^ codes for each patient, n								
Mean	25	14	27	16	26	15	30	15
Maximum	51	68	81	71	82	96	63	66
Minimum	3	3	9	3	6	2	5	4

a Categories of marital status include “single”, “divorces”, “widowed”, and “separated”.

b ICU: intensive care unit.

c *ICD-9*: *International Classification of Diseases, Ninth Revision*.

### Feature Selection

Our experiment’s initial dataset comprised thousands of diagnosis codes intended for predicting cancer diagnosis risk. Aware of some features’ potential redundancy and less informative nature, we did a feature selection process. This involved assessing the relevance and importance of each feature in relation to 4 specific types of cancer. We performed a correlation-based feature selection process to identify a subset of features that were highly correlated with the target cancer outcomes. This was followed by a thorough review of relevant literature and consultation with experts to validate and refine the selected features.

### Framework

In this work, we applied 3 advanced models, penalized LR, RF, and MLP, based on their demonstrated accuracy and robustness in handling high-dimensional datasets. RF and MLP excel at identifying complex, nonlinear interactions among variables without requiring predefined interaction terms. This capability is crucial for analyzing interactions between risk factors and cancer outcomes. Our choice of RF and MLP was determined by a desire to balance complexity with interpretability, as well as to ensure computational efficiency. Both methods are straightforward and offer high interpretability, which makes them excellent foundational models for exploring how different features influence cancer diagnosis risk.

Since the task aimed at forecasting cancer diagnosis risk by considering important and relevant risk factors, we evaluated the efficacy of our methodologies by employing several critical performance metrics: AUC, accuracy, specificity, sensitivity, and the *F*_1_-score for each model. We partitioned the dataset into 3 sections for model development: 70% for training, 10% for validation, and 20% for testing. The model that exhibited the best results on the validation set was further subjected to an in-depth analysis of the test set, using a 3-fold cross-validation technique to calculate its AUC precisely. To enhance our understanding of how our machine learning models contribute to cancer prevention, we also quantified the impact of each feature on the prediction of 4 cancer types. We then ranked these features according to their significance. All statistical analyses and model implementations were coded using Python, with the scikit-learn library serving as the foundation for our predictive framework [39]. To assess the generalizability of the model, we validated its performance using an independent ICU dataset from MIMIC-IV-ED ((Medical Information Mart for Intensive Care), which represents an extended patient population. For each cancer type, we randomly sampled 200 cases and 200 matched controls from MIMIC-IV-ED, ensuring no patient overlap with the MIMIC-III experimental dataset.

To investigate the similarity of features ranking by different cancer types, we applied rank biased overlap (RBO) [40], a similarity measure of 2 ranked lists. The RBO score ranges between 0 and 1, where a higher score indicates greater similarity between the lists. A score of 1 implies perfect overlap, meaning the 2 lists are identical in both order and content. On the other hand, a score of 0 suggests no overlap between the lists.

Mathematically, let $x_{i}$ be the high-dimensional feature input. Let $y_{i}\in \left\{0,1\right\}$ be the corresponding label. $y_{i}=0$ means not affected, and $y_{i}=1$ means affected. Our goal is to learn a predictive function f that best classifies the data. We built 3 state-of-the-art models for 4 cancer types respectively in this study:

- Penalized LR: given M training instances, we considered L1 regularized LR by minimizing the following function: $\sum_{i=1}^{M}-loglogp\left(x^{\left(i\right)};\theta \right)+\beta ||\theta |{|}_{1}$.
- RF [41]: a robust ensemble learning method that constructs multiple decision trees during training to improve prediction accuracy and prevent overfitting, where f is the decision tree as base learners. The RF model was trained by iteratively selecting features from root to leaf nodes and aggregating multiple trees with the weights from a subset of the training instances. The nodes and the weights in the model reflect their importance to the final prediction.
- MLP [42]: a type of artificial neural network that consists of at least 3 layers of nodes: an input layer, one or more hidden layers, and an output layer. Each node, or artificial neuron, in one layer, connects with a certain weight to every node in the following layer, and nodes do not connect within the same layer. The nonlinear activation functions, such as the sigmoid, or Rectified Linear Unit, are applied to the weighted sum of inputs to a neuron, determining its output signal.

To rank the impact on predictive models of the features, relative to all 3 models, we used a permutation importance score to rank all features in the training models for MLP [43]. The scores were defined by the mean decrease in accuracy of the trained model when each feature was permuted.

### Ethical Considerations

MIMIC-III data are the result of a collaboration between Beth Israel Deaconess Medical Center (BIDMC) and Massachusetts Institute of Technology. Data collected at BIDMC as part of routine clinical care are deidentified, transformed, and made available to researchers who have completed training in human research and signed a data use agreement. The Institutional Review Board (HUM00230096) at the BIDMC granted a waiver of informed consent and approved the sharing of the research resource. This study was determined to be exempt from further ethical review. The contributing author, XH, obtained the necessary authorization to access the anonymized dataset and oversaw the meticulous data extraction process.

## Results

### Feature Selection

We conducted a feature selection process to refine thousands of diagnosis codes for predicting cancer diagnosis risk, using correlation-based selection to identify the most relevant features for 4 cancer types. Through this rigorous analysis, we aimed to distill the dataset down to a more manageable and meaningful subset of features. Eventually, we identified 33 features (recategorized into 20 factors for further analysis, Table 2) that emerged as particularly crucial for accurately predicting cancer diagnosis risk. These features were meticulously curated, ensuring that only the most informative and pertinent variables were retained for our predictive models.

**Table 2.** Features selected for predicting cancer diagnosis risks.

Features	Factors
Acidosis	Acidosis
Acute kidney failure, unspecified	Acute kidney failure
Age	Age
Anemia, unspecified	Anemia
Acute posthemorrhagic anemia	Anemia
Depressive disorder, not elsewhere classified	Depressive disorder
Diabetes mellitus without mention of complication, type II or unspecified type, not stated as uncontrolled	Diabetes
Esophageal reflux	Esophageal reflux
Ethnicity	Ethnicity
Gender	Gender
Cardiac complications, not elsewhere classified	Heart disease
Aortocoronary bypass status	Heart disease
Coronary atherosclerosis of native coronary artery	Heart disease
Old myocardial infarction	Heart disease
Congestive heart failure, unspecified	Heart disease
Atrial fibrillation	Heart disease
Subendocardial infarction, initial episode of care	Heart disease
Pure hypercholesterolemia	Hyperlipidemia
Other and unspecified hyperlipidemia	Hyperlipidemia
Unspecified essential hypertension	Hypertension
Other iatrogenic hypotension	Hypotension
Unspecified acquired hypothyroidism	Hypothyroidism
Marital status	Marital status
Religion	Religion
Acute respiratory failure	Respiratory or pulmonary diseases
Unspecified pleural effusion	Respiratory or pulmonary diseases
Pneumonia, organism unspecified	Respiratory or pulmonary diseases
Pneumonitis due to inhalation of food or vomitus	Respiratory or pulmonary diseases
Pulmonary collapse	Respiratory or pulmonary diseases
Chronic airway obstruction, not elsewhere classified	Respiratory or pulmonary diseases
Unspecified septicemia	Sepsis
Personal history of tobacco use	Tobacco use
Urinary tract infection, site not specified	Urinary tract infection (UTI)

### Model Performance

**Figure 2.**
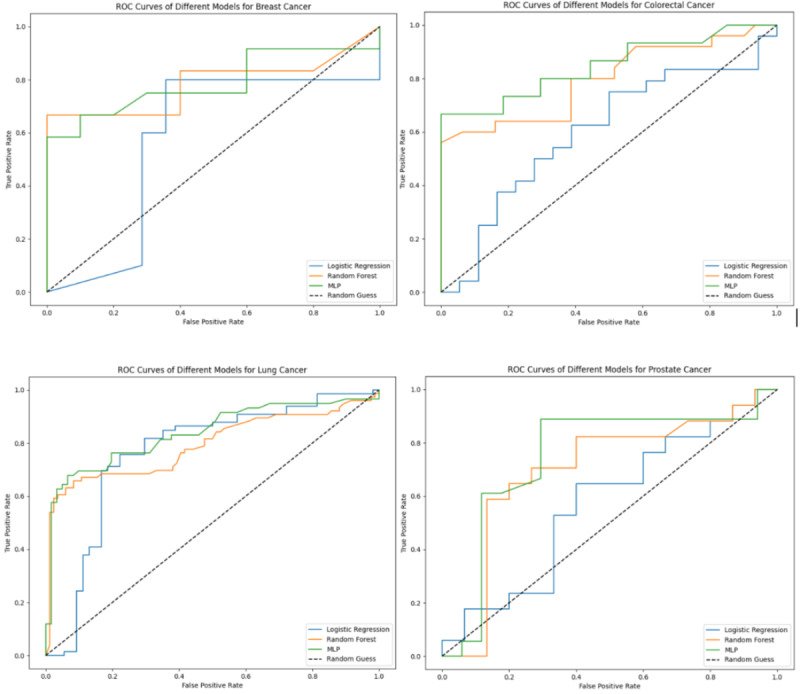
Area under the receiver operating characteristic curve (AUC) performance of the 3 binary classification models (logistic regression [LR], random forest [RF], and multilayer perceptron [MLP]). The figure shows AUC curves of breast cancer, colorectal cancer, lung cancer, and prostate cancer for LR, RF, and MLP, respectively.

For each predicted cancer outcome, we carried out the experiment by predicting cancer using the entire diagnosis history of the patient by building LR, RF, and MLP models. Table 3 illustrates the accuracy, specificity, sensitivity, and *F*_1_-score of these 3 models for breast, colorectal, lung, and prostate cancers. Figure 2 shows the receiver operating characteristic plots of 3 models for 4 types of cancer, respectively. Both Table 3 and Figure 2 show that within the 3 models, MLP performs the best, RF falls in the middle, and LR ranks last. It is worth noting that MLP achieved an AUC of 0.78 for breast cancer, 0.76 for colorectal cancer, 0.84 for lung cancer, and 0.78 for prostate cancer, demonstrating a higher AUC over traditional risk factor-based models and a statistically significant superiority over random chance. The underwhelming results from the LR model led us to investigate the complexity of risk factors for prediction. Compared with LR, MLP reveals the intricate, nonlinear associations between risk factors and the likelihood of cancer, offering meaningful insights into the collective influence of these risk factors on cancer diagnosis risk.

**Table 3.** Comparison of model performance across 4 types of cancer on Medical Information Mart for Intensive Care (MIMIC)–III.

	Breast cancer			Colorectal cancer			Lung cancer			Prostate cancer		
	LR^a^	RF^b^	MLP^c^	LR	RF	MLP	LR	RF	MLP	LR	RF	MLP
Accuracy	0.56	0.73	0.78	0.60	0.70	0.76	0.74	0.80	0.83	0.59	0.72	0.78
Specificity	0.45	0.70	0.80	0.67	0.61	0.81	0.61	0.92	0.87	0.53	0.80	0.84
Sensitivity	0.71	0.75	0.75	0.54	0.80	0.73	0.85	0.68	0.80	0.65	0.65	0.72
*F*_1_-score	0.56	0.75	0.75	0.60	0.70	0.79	0.78	0.78	0.84	0.63	0.71	0.76

a LR: logistic regression.

b RF: random forest.

c MLP: multilayer perceptron.

Additionally, Table S2 in Multimedia Appendix 1 presents the AUC, accuracy, specificity, sensitivity, and *F*_1_-score for the 3 models across breast, colorectal, lung, and prostate cancers. Among the models evaluated, MLP demonstrated the highest performance, achieving an AUC of 0.88 for breast cancer, 0.83 for colorectal cancer, 0.90 for lung cancer, and 0.85 for prostate cancer.

### Feature Importance Analysis

**Figure 3.**
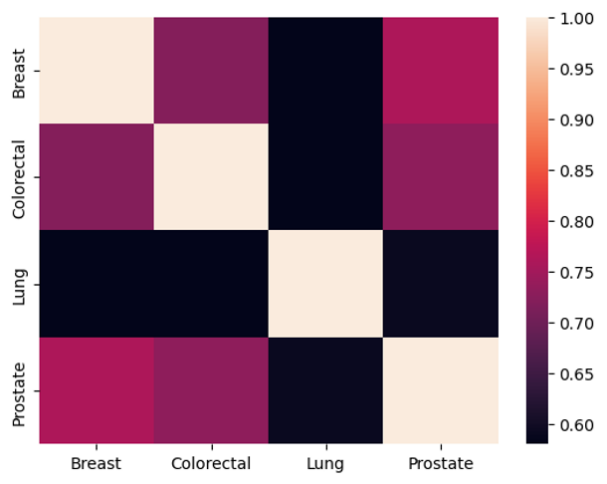
Rank biased overlap similarity score of risk factors for 4 cancer types. A high value represents high similarity, and a low value represents low similarity of risk factor ranks between 2 cancer types.

We analyzed the feature importance for each cancer type further to investigate the potential impact of risk factors on cancer. Tables4  5 present the feature importance analysis of RF and MLP, showcasing the top-ranked risk factors for each type of cancer. The ranks of these factors were relatively different by model and cancer type, although some consistency can be observed across cancer types. Age emerged as the top risk factor across all 4 types of cancer; race/ethnicity ranked among the top 10 factors for all cancers from all models except for the RF-based lung cancer and prostate cancer models; gender was ranked among the top 10 in MLP-based models but not in any RF-based models; marital status and religion were presented for some types of cancer in some of the models; and tobacco use as an important factor for patients with lung and prostate cancer exclusively. However, all these demographic risk factors were included in the top 20 factors for all cancer types (Table S3 in Multimedia Appendix 1). Similarly, RF-based models identified hypertension, heart diseases, respiratory/pulmonary diseases, and acute kidney failure as the common top risk factors for all types of cancers, while MLP-based models highlighted hyperlipidemia, diabetes, depressive disorder, and heart diseases. We calculated the odds ratio (OR) for each highlighted feature to assess its association with overall cancer diagnosis risk across 4 cancer types. The results indicated that hyperlipidemia had an OR of 1.14 (95% CI 1.11‐1.17; *P*<.001), while diabetes showed a stronger association with an OR of 1.34 (95% CI 1.29‐1.39; *P*<.01). Similarly, depressive disorders were linked to an OR of 1.11 (95% CI 1.06‐1.16 *P*<.01), and heart diseases exhibited the highest association with an OR of 1.42 (95% CI 1.32‐1.52; *P*<.01). Last, anemia was also significantly associated with cancer diagnosis risk, with an OR of 1.22 (95% CI 1.14‐1.30; *P*<.01). These findings suggest a statistically significant relationship between these conditions and an increased risk of developing these 4 types of cancer. In MLP-based models, respiratory/pulmonary diseases and acute kidney failure were only presented as the top 10 for lung cancer. Both RF and MLP-based models pinpointed anemia as the top risk for breast cancer. Figure 3 shows the RBO similarity scores of risk factors for 4 types of cancer according to MLP-based models. Low similarity scores are presented between lung cancer and any other 3 cancer types, all around 0.58, suggesting distinct patterns of risk factors associated with lung cancer. Risk factors for breast and prostate cancers show the most similar ranking with an RBO similarity score of 0.76. A moderate similarity score between colorectal and breast cancers is about the same as the score between colorectal and prostate cancer, both around 0.70.

**Table 4.** Top-10 ranked features generated across 4 different cancer types in random forest.

Ranking	Breast cancer	Colorectal cancer	Lung cancer	Prostate cancer
1	Age	Age	Age	Age
2	Hypertension	Respiratory or pulmonary diseases^a^	Hypertension	Hypertension
3	Religion	Hypertension	Religion	Religion
4	Marital status	Acute kidney failure	Hyperlipidemia	Heart diseases^c^
5	Respiratory or pulmonary diseases	Diabetes	Heart diseases	Marital status
6	Heart diseases	Heart diseases	Acute kidney failure	UTI^c^
7	Race or ethnicity	Hyperlipidemia	UTI	Respiratory or pulmonary diseases
8	Depressive disorders	Race or ethnicity	Respiratory or pulmonary diseases	Anemia
9	Acute kidney failure	Religion	Marital status	Hyperthyroidism
10	Anemia	Acidosis	Anemia	Diabetes

a Respiratory or pulmonary diseases include pneumonia, acute respiratory failure, chronic airway obstruction, and other respiratory or pulmonary complications.

b Heart diseases include atrial fibrillation, myocardial infarction, congestive heart failure, coronary atherosclerosis, and other cardiac complications.

c UTI: urinary tract infection.

**Table 5.** Top-10 ranked features generated across 4 different cancer types in multilayer perceptron.

Ranking	Breast cancer	Colorectal cancer	Lung cancer	Prostate cancer
1	Age	Age	Tobacco use	Age
2	Gender	Diabetes	Age	Gender
3	Hyperlipidemia	Anemia	Respiratory or pulmonary diseases^a^	Race or ethnicity
4	Heart diseases^b^	Acidosis	Gender	Tobacco use
5	Race or ethnicity	Hyperlipidemia	Race or ethnicity	Diabetes
6	Marital status	Sepsis	Diabetes	Hyperlipidemia
7	Depressive disorder	Gender	Hyperlipidemia	Heart diseases
8	Religion	Race or ethnicity	Hypertension	Marital status
9	Anemia	Marital status	Heart diseases	Religion
10	Hypothyroidism	Depressive disorder	Acute kidney failure	Depressive disorder

a Respiratory or pulmonary diseases include pneumonia, acute respiratory failure, chronic airway obstruction, and other respiratory or pulmonary complications.

b Heart diseases include atrial fibrillation, myocardial infarction, congestive heart failure, coronary atherosclerosis, and other cardiac complications.

## Discussion

### Principal Findings

This study used comprehensive patient diagnosis histories to evaluate the association between key risk factors and cancer outcomes and identify risk factor patterns across different cancer types using penalized LR, RF, and MLP models. The analysis identified the top-ranking risk factors, including nontraditional risk factors such as the diagnosis of hyperlipidemia, diabetes, depressive disorders, heart diseases, and anemia, in addition to demographic factors such as age, sex, race/ethnicity, for the most prevalent 4 types of cancer, including breast, colorectal, lung, and prostate cancers. The model performance evaluation revealed the valuable potential of neural network-based models, especially MLPs, in oncology for predicting cancer diagnosis risks across cancer types. MLPs exhibit a strong capability to model complex, nonlinear interactions among diverse risk factors, making them potentially valuable tools for identifying patterns in cancer diagnosis risk and informing early detection strategies. However, their application in clinical interventions should be guided by a solid scientific rationale and supported by pathological models that explain the role of these risk factors in disease development. Additionally, validation across different cohorts and, ideally, prospective studies are necessary to ensure their reliability and clinical utility. This advantage is particularly important given the model’s capacity to integrate and interpret the intricate relationships between clinical factors present in EHRs. In contrast to simpler models like LR, which struggle with the multidimensional nature of risk factors on cancer diagnosis in many cases, MLPs offer a more detailed and comprehensive analysis, enhancing our understanding of how these factors collectively impact cancer diagnosis risk and improving the precision of preventive strategies in clinical settings. Last, this study does not aim to establish causal inference but rather to examine significant overlapping risk factors that may contribute to cancer diagnosis risk, particularly those observed in patients with other medical conditions. While these diagnoses are not independent causal determinants of cancer, their presence may be associated with an increased risk. Careful consideration of these associations is essential for a comprehensive understanding of cancer risk factors and their potential interactions.

### Comparison to Prior Work

Prior cancer risk prediction models usually focus on lifestyle factors like smoking, diet, alcohol consumption, physical activity, and sun exposure as key variables [44-46]. Some models have also incorporated genetic risk factors [47,48]. However, many of these models reported less optimal performance, such as a high specificity but low sensitivity [46] or a low AUC of around 0.65 [48]. Chronic diseases are often overlooked as risk factors for cancer, and they are not often targeted in cancer prevention strategies. The association between some of these diseases and cancers may partly be due to shared risk factors, such as aging, obesity, diet, and physical inactivity. However, they can also be independent risk factors for cancer. For example, diabetes mellitus has been identified as an independent risk factor for colon and rectal cancer in a meta-analysis of studies that either controlled for smoking and obesity, or smoking, obesity, and physical exercise [49]. As nontraditional risk factors, the influence of certain chronic conditions on cancer has been brought to researchers’ attention in the past decade. A prospective cohort study with 405,878 participants followed for an average of 8.7 years demonstrated that 8 common chronic diseases accounted for more than 20% of cancer risk, which are comparable to 5 major lifestyle factors, such as smoking and lack of physical activity [16]. These 8 chronic diseases or markers included blood pressure, total cholesterol, heart rate, diabetes, proteinuria, glomerular filtration rate, pulmonary disease, and gouty arthritis marker [16]. However, as these diseases or markers were pre-selected by the researchers based on their disease burden worldwide, some other essential influential conditions might be missed. Our models confirmed most of these 8 diseases as the top-ranking risk factors. Additionally, some new conditions were revealed in our models among the top 10 factors for 4 types of cancer, such as depressive disorder, anemia, hypothyroidism, sepsis, urinary tract infection, and acidosis, which encourages further exploration. Certainly, some of these diagnoses may be directly related to the cancer itself. For example, anemia is a common symptom of metastatic breast cancer and a side effect of chemotherapy [50]. In addition, sepsis and colorectal cancer have demonstrated a complex relationship and may have shared pathophysiological traits and potential bacterial associations reported by the literature [51]. Notably, tobacco use and respiratory/pulmonary diseases emerged as pivotal risk factors, specifically for lung cancer, which is not surprising based on our knowledge in the field. Diabetes and anemia were highlighted as significant risk factors for colorectal cancer, which is congruent with the literature [52,53]. Iron deficiency has been recognized long-term as an independent predictor of colorectal cancer, which may be due to chronic blood loss from the gastrointestinal tract and the inflammation associated with malignancy [54,55]. These conditions could have shared risk factors with cancer. However, emerging evidence implies that they may have more complicated relationships, including shared pathophysiological mechanisms that need further exploration [56]. Moreover, cancer prevention strategies should consider the impact of comorbid conditions on the incidence of cancer and particularly their joint impact on cancer risks [53].

Understanding the relationships between various risk factors and cancer diagnosis risk is pivotal for the early detection and prevention of cancer. In this context, our feature importance analysis using RF and MLP models pinpointed critical risk factors for different cancer types and explored patterns of these risk factors across various cancers. Although the ranks of risk factors for cancers were slightly different by the RF and MLP-based models, similar patterns were presented among the top 10 factors (Tables4  5), which are interpretable and supported by the literature. Both models highlighted age as the predominant risk factor across all 4 types of cancer, which is evident that as age increases, the incidence rates for cancer overall climb steadily, and alongside age, demographic variables such as gender, race/ethnicity, marital status, and religion emerged within the top 10 features [57]. Racial/ethnic disparities in cancer incidence and outcomes are well-known. Employing culturally tailored community awareness and education programs may increase cancer screening to improve early-stage diagnoses and modify risk behaviors for cancer prevention [58]. Although there may not be existing evidence to confirm that marital status is an independent risk factor for cancer, observational studies demonstrate that married status is associated with reduced risk of cancer-specific and all-cause mortality [59,60]. Religion and spirituality are important in patient cancer care, and specifically, a systematic review suggests a positive association between religious attendance and cancer screening use [61]. Our models not only confirmed the significance of these risk factors for each cancer type but also our RF-based model facilitated an interpretable analysis, allowing us to clearly rank the significance of each risk factor, while the MLP-based model provided deeper insights into complex, nonlinear interactions among the risk factors. This approach enriches our understanding of how specific risk factors influence cancer diagnosis, enhancing the potential for developing tailored intervention strategies that address the unique risk profiles associated with different cancer types and potentially shared risk patterns across prevalent cancer types.

The analysis of the similarity among risk factors for the diagnosis of 4 types of cancer also revealed interesting findings. As breast and prostate cancer are both hormone-dependent cancers, it is understandable that their importance-ranked risk factors share a high level of similarity. However, lung cancer had more unique ranked risk factors than other types of cancer, which may be because lung cancer is more sensitive to environmental risk factor exposure. The findings from our analysis underscore the shared risk factors and heterogeneous nature of cancer and highlight the importance of considering unique risk profiles for different cancer types. This also urges us to address the fundamental mechanism of risk factors leading to cancers. Such insights are crucial for developing tailored prevention strategies, optimizing screening protocols, and informing personalized treatment approaches to mitigate the burden of lung cancer and improve patient outcomes.

### Limitations

First, the use of the MIMIC-III dataset in this study on explainable machine learning for cancer risk prediction presents certain limitations that may impact the generalizability of the findings, Since the data are derived from ICU patient records, it primarily represents individuals with severe conditions, and the available *ICD* codes may not fully capture disease complexity, potentially leading to incomplete representations of patient conditions. Additionally, the limited sample size for patients with cancer may impact predictive performance and increase the risk of overfitting. Both limitations may affect the generalizability of the findings. To enhance the robustness of future research, integrating more recent and varied data sources and validating findings across different cohorts are essential steps. Second, one limitation comes from the application of explainable machine learning models for cancer risk prediction. Employing advanced techniques like penalized LR, RF, and MLP, this research seeks to optimize predictive accuracy. However, each model inherently embodies trade-offs: while more complex models, such as multi-layer perceptron, may enhance performance, they often compromise on interpretability. This presents significant challenges in clinical settings, where understanding the reasoning behind model predictions is crucial for acceptance and trust by medical practitioners. Third, another limitation of this study arises from the inherent nature of machine learning models, which are primarily designed to detect correlations in data and associations between features and the outcome rather than establish causal relationships. These models rely on the quality and comprehensiveness of the input data, and while they can reveal significant associative patterns, they do not focus on differentiating whether the associations observed are causal. Meanwhile, given the limited availability of patient lifestyle and socioeconomic information, additional factors related to social determinants of health, such as socioeconomic status, employment, and family size, can be considered as potential confounders within the model for future improvement. To address all the above, future work should integrate causal inference frameworks to validate the relationships suggested by the machine learning predictions and provide insights into underlying mechanisms.

### Conclusions

In conclusion, our study established a predictive framework using EHR data to assess the association between risk factors and cancer outcomes using explainable ML models across major cancer types. We reported critical nontraditional chronic condition risk factors in addition to common demographic risk factors and outlined distinct patterns for each of the 4 cancer types studied. Additionally, we explored the similarities and differences in risk factor patterns across these cancers. These insights contribute to a better understanding of cancer risk profiles and benefit in improving cancer diagnosis and risk monitoring, offering supportive guidance for clinical decision-making.

## Supplementary material

10.2196/62833 Multimedia Appendix 1 Supplementary tables on the *ICD-9* codes description, model performance, and top 20 ranked features for the 4 cancer types

## Abbreviations

AUC: area under the receiver operating characteristic curve
BIDMC: Beth Israel Deaconess Medical Center
EHR: electronic health record
*ICD-9*: *International Classification of Diseases, Ninth Revision*
ICU: intensive care unit
LR: logistic regression
MIMIC: Medical Information Mart for Intensive Care
MLP: multilayer perceptron
OR: odds ratio
RBO: rank biased overlap
RF: random forest
