# Supplementary material for: Association Between Risk Factors and Major Cancers: Explainable Machine Learning Approach
Source: JMIR Cancer. 2025 May 2;11:e62833. doi: 10.2196/62833 (PMC12064211; doi:10.2196/62833)
Supplement: Multimedia Appendix 1 [file cancer-v11-e62833-s001.docx]

**Appendix:**

Table S1. Description of ICD-9 Disease Codes

| Cancer Type | ICD-9 Code* | Description |
| --- | --- | --- |
| Breast cancer | 174 | Malignant neoplasm of female breast |
|  | 175 | Malignant neoplasm of male breast |
| Colorectal cancer | 153 | Malignant neoplasm of colon |
|  | 154 | Malignant neoplasm of rectum rectosigmoid junction and anus |
| Lung cancer | 162.3 | Malignant neoplasm of upper lobe, bronchus or lung |
|  | 162.4 | Malignant neoplasm of middle lobe, bronchus or lung |
|  | 162.5 | Malignant neoplasm of lower lobe, bronchus or lung |
|  | 162.8 | Malignant neoplasm of other parts of bronchus or lung |
|  | 162.9 | Malignant neoplasm of bronchus and lung, unspecified |
| Prostate cancer | 185 | Malignant neoplasm of prostate |

*: The patient is considered cancer diagnosed if one of the ICD-9 Code from specific type of cancers occur during his/her visits

Table S2. Comparison of model performance across four types of cancer on MIMIC-IV sampled data.

|  | Breast cancer | | | Colorectal cancer | | | Lung cancer | | | Prostate cancer | | |
| --- | --- | --- | --- | --- | --- | --- | --- | --- | --- | --- | --- | --- |
|  | LR | RF | MLP | LR | RF | MLP | LR | RF | MLP | LR | RF | MLP |
| Accuracy | 0.73 | 0.76 | 0.84 | 0.71 | 0.72 | 0.83 | 0.72 | 0.81 | 0.88 | 0.62 | 0.72 | 0.80 |
| Specificity | 0.69 | 0.81 | 0.85 | 0.58 | 0.70 | 0.89 | 0.70 | 0.74 | 0.76 | 0.70 | 0.80 | 0.83 |
| Sensitivity | 0.78 | 0.80 | 0.83 | 0.72 | 0.78 | 0.80 | 0.79 | 0.81 | 0.88 | 0.66 | 0.68 | 0.87 |
| F1-score | 0.71 | 0.74 | 0.84 | 0.71 | 0.75 | 0.82 | 0.72 | 0.77 | 0.83 | 0.67 | 0.74 | 0.86 |
| **AUC** | **0.81** | **0.84** | **0.88** | **0.72** | **0.75** | **0.83** | **0.80** | **0.84** | **0.90** | **0.63** | **0.73** | **0.85** |

Table S3. Top-20 ranked features generated across four different cancer types.

| **Model: RF** | | | | |
| --- | --- | --- | --- | --- |
| Ranking | Breast Cancer | Colorectal Cancer | Lung Cancer | Prostate Cancer |
| 1 | Age | Age | Age | Age |
| 2 | Hypertension | Respiratory/Pulmonary diseases | Hypertension | Hypertension |
| 3 | Religion | Hypertension | Religion | Religion |
| 4 | Marital status | Acute kidney failure | Hyperlipidemia | Heart Diseases |
| 5 | Respiratory/Pulmonary diseases* | Diabetes | Heart diseases | Marital status |
| 6 | Heart diseases** | Heart diseases | Acute kidney failure | UTI |
| 7 | Race/Ethnicity | Hyperlipidemia | UTI | Respiratory/Pulmonary |
| 8 | Depressive disorders | Race/Ethnicity | Respiratory/Pulmonary | Anemia |
| 9 | Acute kidney failure | Religion | Marital status | hyperthyroidism |
| 10 | Anemia | Acidosis | Sepsis | Diabetes |
| 11 | Esophageal Reflux | Marital status | Anemia | Race/ethnicity |
| 12 | UTI | Hyperthyroidism | Diabetes | Acute kidney failure |
| 13 | Hyperlipidemia | Tobacco use | Gender | Hyperlipidemia |
| 14 | Acidosis | Anemia | Race/Ethnicity | Sepsis |
| 15 | Gender | Gender | Acidosis | Esophageal reflux |
| 16 | Hyperthyroidism | UTI | Hyperthyroidism | Acidosis |
| 17 | Tobacco use | Esophageal reflux | Esophageal reflux | Depressive disorders |
| 18 | Sepsis | Hypotension | Depressive disorders | Tobacco use |
| 19 | Hypotension | Depressive disorders | Tobacco use | Hypotension |
| 20 | Diabetes | Sepsis | Hypotension | Gender |
| **Model: MLP** | | | | |
| Ranking | Breast Cancer | Colorectal Cancer | Lung Cancer | Prostate Cancer |
| 1 | Age | Age | Tobacco use | Age |
| 2 | Gender | Diabetes | Age | Gender |
| 3 | Hyperlipidemia | Anemia | Respiratory/Pulmonary diseases | Race/Ethnicity |
| 4 | Heart diseases | Acidosis | Gender | Tobacco use |
| 5 | Race/Ethnicity | Hyperlipidemia | Race/Ethnicity | Diabetes |
| 6 | Marital status | Sepsis | Diabetes | Hyperlipidemia |
| 7 | depressive disorder | Gender | Hyperlipidemia | Heart diseases |
| 8 | Religion | Race/Ethnicity | Hypertension | Marital status |
| 9 | Anemia | Marital status | Heart diseases | Religion |
| 10 | Hypothyroidism | Depressive disorder | Acute kidney failure | Depressive disorder |
| 11 | Diabetes | Religion | Anemia | Hypothyroidism |
| 12 | Hypertension | Hypertension | Esophageal reflux | Anemia |
| 13 | Respiratory/Pulmonary diseases | Respiratory/Rulmonary diseases | UTI | Hypertension |
| 14 | Acute kidney failure | Heart diseases | Acidosis | Respiratory/Pulmonary diseases |
| 15 | Esophageal reflux | Acute kidney failure | Hypotension | Acute kidney failure |
| 16 | UTI | Tobacco use | Sepsis | esophageal reflux |
| 17 | Acidosis | Esophageal reflux | Depressive disorder | UTI |
| 18 | Tobacco use | UTI | Hypothyroidism | Acidosis |
| 19 | hypotension | hypotension | Marital status | Hypotension |
| 20 | sepsis | hypothyroidism | Religion | Sepsis |

*: Respiratory/Pulmonary diseases include pneumonia, acute respiratory failure, chronic airway obstruction, and other respiratory or pulmonary complications.

**: Heart diseases include atrial fibrillation, myocardial infarction, congestive heart failure, coronary atherosclerosis, and other cardiac complications.
